# Dietetics

**Published:** 1905-07-15

**Authors:** 


### DIETETICS.

**Euphagia.**—This is the name given by Einhorn [1] to the art of eating properly. The points to be observed are: the body should have previous work, not, however, carried to the point of exhaustion, and subsequent rest. Meals should be taken in pleasant company and enlivened by conversation, owing to the important part played by the higher cerebral centres in stimulating digestion. Very important is the due mastication of food, by which the coarser particles are broken up and time allowed for the action of the saliva. Water should be taken with meals, as it increases the appetite and enjoyment of food, and prevents the food from being consumed too hot. The two errors to be avoided are hasty eating (tachyphagia) and slow eating (bradyphagia). Hasty eating is very common, and results in imperfect preparation of the food for gastric digestion, the passage of coarse irritating particles into the intestine, and mal-digestion of starch. It also results in too great a quantity of food being consumed, and often allows of food being swallowed while too hot. A host of digestive troubles results. The opposite error is rare and only observed in neurasthenics. It results in practical starvation and a condition of inability to swallow anything. Einhorn cites two cases: one patient took half an hour to consume a glass of milk, and the other 20 minutes for a plate of soup and 25 minutes for a cup of milk. This condition requires active moral treatment. Patients must be made to take their meals with others and finish them in a reasonable time. Bromides and valerian are occasionally useful.

**Underfeeding.**—The criterion of underfeeding is not merely body weight but metabolic equilibrium, as is pointed out by Stern.[2] Those who are really underfed cannot be restored to health by any stuffing process; their system must first be prepared to elaborate and assimilate foodstuffs properly. This is well seen in the so-called "wasting diseases," such as tuberculosis, when actual wasting does not occur until metabolic processes begin to go wrong. The point to keep in view in treating really underfed persons is the proper adaptation of the diet to the individual, apart from the mere disease from which he is suffering. The diet should produce no unpleasant symptoms, should not be bulky, should not tax the digestive powers, should be rich in heat-producing and tissue-saving substances. The yolk of hens' eggs is a most valuable article for this purpose. Yolk, raw or half raw, is easily digested, nearly completely assimilated, contains a useful diastatic ferment, and efficiently stimulates gastric secretion. The contained lecithin may tend to restore nervous force, and the heat value of the fatty substances is high, an average egg yolk producing 50 calories. Moreover, it is only the white of egg which is badly tolerated by those persons who are said to be unable to digest eggs: it yields sulphuretted hydrogen and ammonia, and is, owing to its coagulability, a solid food, liable to remain long in the stomach when motor insufficiency is present. Yolks may be given without other food, in which case a slight loss of nitrogen occurs, or added to other food in lieu of other forms of fat. The latter method is very useful in diabetes, as it lessens the glycosuria and stops the production of acetone bodies. In non-diabetic cases the determination of the remainder of the diet is a very important point, as faulty directions as to proteid and starchy foods retard or suppress the beneficial action of the yolk. Each case must be carefully considered and individually treated. The yolks may be given in tea, coffee, milk, or dilute spirits, or cooked with various articles of diet. At first they should be the only form of fat given; later on limited amounts of other fats may be given on certain days. The writer gives an illustrative *ménu* for a consumptive weighing 110 lb. requiring 1,750 calories in 24 hours. Fifteen eggs' yolks are given in various ways, in addition to a proteid and carbohydrate diet. The latter should be as varied as possible, and a due amount of salt should always be added to the yolk diet.

**Boiled Milk and Gruel.**—In spite of the fact that bacteria present in milk are removed by the process of sterilisation, Spolverini[3] considers sterilised milk an improper food for infants. The toxins and dead bacterial bodies are still present; the carbonic acid, which stimulates gastric digestion, is removed; the lime and free phosphoric acid are diminished, and the insoluble calcium phosphate is increased; the citric acid (antiscorbutic) is precipitated, and a large part of the licithin and nucleon destroyed; the casein is changed to an unabsorbable substance, and the soluble albumen is coagulated; the fat globules are rendered less easy of absorption; and, lastly, the ferments, which are important immunising and antitoxic substances, are destroyed. Thus, children fed on sterilised milk are apt to be pale, flabby, and slightly rickety, with a loss of resistance to disease of various kinds. The use of gruels as diluents to milk has long been advocated by various authorities: they are easily retained and absorbed at the expense of a minimum amount of energy by the organism. Chapin[4] has devised a method for standardising these useful substances on the same principle as milk mixtures are standardised by Rotch and others. By using definite weights of cereals and definite degrees of dilution, gruels containing a known percentage of proteid and carbohydrate may be obtained. The fat and mineral matter may be disregarded, as they are so small in quantity. In making these gruels it is important that the cereal used should be thoroughly cooked to break up the starch granules and that the product should not be strained. To facilitate the process Chapin has estimated the weight of different cereals contained in various domestic measures, such as the tablespoon and the 16-ounce measuring glass. He finds that whereas plain gruels cannot be made to contain much more than two ounces of cereal to the quart, dextrinised gruels can be made as strong as eight ounces to the quart. Though not considering that very elaborate percentage feeding can accomplish what is sometimes claimed for it, Chapin thinks the principle, if not carried too far, highly advantageous. In persistent vomiting and in the enfeebled digestive states seen in typhoid and other fevers, high percentage gruels are most useful. It is an error to consider that vegetable proteids are not readily digested and absorbed; 98 per cent. of the proteid in white bread is digested and the proteid of oatmeal is almost as thoroughly digested as that of meat. The cellulose envelope which is the real cause of malabsorption of proteid derived from cereals, may be removed by proper cooking. The cereal, moreover, should not be coarse in texture, or it will pass too quickly through the digestive tract.

[1] Med. Rec., Jan. 7, 1905, p. 9. [2] Ibid., Dec. 31, 1904, p. 1049. [3] Ibid., Dec. 3, 1904, p. 913. [4] Ibid., Feb. 18, 1905, p. 246.

(*To be continued.*)

